# Empiric Recurrence Risk Estimates for Chronic Tic Disorders: Implications for Genetic Counseling

**DOI:** 10.3389/fneur.2020.00770

**Published:** 2020-08-11

**Authors:** Gary A. Heiman, Jessica Rispoli, Christine Seymour, James F. Leckman, Robert A. King, Thomas V. Fernandez

**Affiliations:** ^1^Department of Genetics and the Human Genetics Institute of New Jersey, Rutgers, The State University of New Jersey, Piscataway, NJ, United States; ^2^Child Study Center, Yale University School of Medicine, New Haven, CT, United States; ^3^Department of Psychiatry, Yale University School of Medicine, New Haven, CT, United States

**Keywords:** Tourette disorder, chronic tic disorders, genetic counseling, recurrence risk estimate, genetic

## Abstract

**Background:** Tourette disorder (TD) and other chronic tic disorders are neurodevelopmental/neuropsychiatric disorders characterized by motor and/or vocal tics. Family studies indicate that TD strongly aggregates within families and that other chronic tic disorders are biologically related such that studies typically combine them into any chronic tic disorder (CTD). Because of stigma, bullying, and comorbidity with other neuropsychiatric disorders, CTDs can severely impact the quality of life of individuals with these disorders.

**Objectives:** The genetic architecture of CTDs is complex and heterogeneous, involving a myriad of genetic variants. Thus, providing familial recurrence risks is based on empirical recurrence risk estimates rather than genetic testing. Because empiric recurrence risks for CTDs have not been published, the purpose of this study is to calculate and report these recurrence risks estimates.

**Methods:** Based on population prevalence and increased risk to different relatives from a large population-based family study, we calculated the empiric recurrent risk estimate for each relative type (full sibling, parents, offspring, all first-degree, and all second-degree).

**Results:** The recurrence risk estimate for CTDs in first-degree relatives is 29.9% [95% confidence interval (CI) = 23.2–38.5%]. The risk is higher in males, 33.7% (95% CI = 26.2–43.3%), than females, 24.3% (95% CI = 18.9–31.3%).

**Conclusions:** Given the complex, heterogeneous genetic architecture of CTDs, individuals concerned about recurrence risk should be referred to genetic counseling. Such counseling should include discussion of the derivation and limitations of these empiric recurrence risk estimates, including the upper and lower limits of the range of risk.

## Introduction

Tourette and other chronic tic disorders are neurodevelopmental/neuropsychiatric disorders characterized by motor and/or vocal tics that make their appearance before the age of 18 years. While the diagnostic criteria for Tourette disorder (TD) require the presence of multiple motor and at least one vocal tic over at least 1 year, the criteria for chronic motor tic (CMTD) or chronic vocal tic (CVTD) disorders require at least 1 year of having either motor or vocal tics, respectively, but not both, during the individual's lifetime (1, 2). Tic severity symptoms characteristically wax and wane throughout the day, weeks, months, and years (3). The 95% lifetime prevalence confidence interval (CI) of TD ranges from 0.32 to 0.85% (4), and combined, the prevalence CI of all chronic tic disorders (CTDs) ranges from 0.92 to 2.83% (5). Chronic tic disorders are found across most ethnic groups worldwide and are more prevalent in males than females (3–4:1) (6, 7). While follow-up studies suggest a third of individuals report tic resolution into adulthood (8), objective videotape evaluations show that nonetheless some of these individuals still manifest tics (9). Over and above their psychosocial sequelae such as stigma or bullying, CTDs can severely impact quality of life because of a high comorbidity rate with other neuropsychiatric disorders including obsessive–compulsive disorder (OCD, 50.0%) and attention-deficit/hyperactivity disorder (ADHD, 54.3%) (6, 10, 11).

Family studies consistently show that TD strongly aggregates within families and that TD, CMTD, and CVTD are biologically sufficiently related that genetic studies typically combine them into a category of any CTD rather than analyzing them separately (6, 7, 11–16). Heritability studies clearly indicate a genetic contribution to the etiology of CTDs, with estimates ranging from 25 to 50% in twin studies (13, 15, 17) to 77% in a population-based familial-clustering study (18). Thus, CTDs are among the most heritable neuropsychiatric conditions (18). The relationship between tic severity and genetic predisposition in these studies is mixed. While proband severity is not associated with the number of affected relatives (19), proband severity is associated with having at least one affected relative (i.e., positive family history) (20). However, tic severity has low heritability (21) and varies considerably within families (22, 23). There is little evidence of assortative mating among individuals with tic disorders (24). However, when present, bilineal transmission has been associated with a higher level of tic severity in the offspring (24). In addition, multiple studies also indicate that OCD is genetically related to CTDs (11, 13, 25, 26) with both shared and distinct genetic risk factors (27).

Because family and twin studies show that CTDs are familial and have, at least in part, a genetic causation, molecular genetic studies have been conducted using different study designs (28). Initially, when it was thought that highly penetrant mutations were necessary and sufficient to cause CTD, linkage studies were conducted using large multiplex families. While these studies often had intriguing initial findings, they could not be replicated or localized to a distinct genetic locus (7, 12). Later, the “common variant–common disease” hypothesis and the “rare variant–common disease” hypothesis paradigms led, respectively, to genome-wide association studies (GWASs) (16, 29) and whole-exome sequencing (WES) studies (14, 30). In the GWASs of CTDs, only one single-nucleotide polymorphism surpassed the genome-wide significance threshold, but this finding was not replicated in an independent cohort (16). However, polygenic risk scores, based on GWAS results, are able to predict tic disorders in independent samples (16, 31). Two recent WES studies, searching for *de novo* damaging variants (variants predicted to disrupt protein function or probably-damaging missense variants), have yielded important insights into the genetic architecture of CTDs (14, 30). First, they estimate 400–500 genes that increase risk for CTDs when mutated in the form of a damaging high-penetrance variant. Second, 10–12% of CTDs are due to *de novo* damaging variants, including *de novo* copy number variants (CNVs). While *de novo* in one individual, these individuals can subsequently pass the variants on to their offspring. Finally, some of the risk genes found so far in WES studies are involved in cell polarity, suggesting that CTDs may be caused by a disruption in neurons arriving at the correct location and making correct connections during neurodevelopment. Taken together, these molecular genetic studies suggest that CTDs are caused by variants in many different genes, some with rare damaging high-penetrance variants, including CNVs, which may interact with a background genetic risk from common low-penetrance variants. Additionally, CTDs may be caused by an additive effect of common low-penetrance variants in many genes (i.e., polygenic), which may or may not overlap with the same genes that harbor rare high-penetrance variants. Future studies may also document the “co-action” of specific gene variants (32). Specific gene variants may also interact with environmental factors such as prenatal maternal smoking (33).

Given the complex and heterogeneous genetic architecture of CTDs, providing familial recurrence risks is challenging. Currently, there are no genetic tests available to determine individualized familial recurrence risk to relatives. Instead, available recurrence risks are empirical estimations based on results from population prevalence and family studies rather than a single risk based on Mendelian inheritance. Because these studies provide risk ranges (i.e., CIs), this makes providing empiric recurrence risk to at-risk individuals even more challenging (34, 35). The purpose of this article is to provide the empirical recurrence risk estimates for CTDs for different familial relationships, as these recurrence risks have not been previously reported. The process of how to optimally present these empiric risk estimates to individuals seeking genetic counseling is beyond the scope of this article and can be found elsewhere (35). However, in the discussion, we present the implications for genetic counseling for CTDs, based on current knowledge of genetic architecture and from empiric recurrence estimates.

## Materials and Methods

### General Population Prevalence of CTDs

There are two published meta-analyses of the general population lifetime prevalence of TD (4, 5). One of these (5) provides estimates for TD and all CTDs combined. We used the CTDs general population prevalence data from this study (5) to calculate the empiric recurrence risk estimates. This meta-analysis found an overall general population prevalence for CTDs of 1.6% (95% CI = 0.9–2.8) with a higher prevalence in males (1.8%; 95% CI = 0.7–4.4%) than females (1.3%; 95% CI = 0.8–2.0%) (5).

### Family Study Data: Increased Risk of CTDs in Relatives

Despite many family studies [for a review, see (12)], there is no published meta-analysis of increased risk of CTDs in relatives. We based our calculation of empiric recurrence risk on a recent family study because it is population-based, it has the largest sample size (*n* = 4,826), and it includes all CTDs rather than only TD (18).

### Calculation of Empiric Recurrence Risk Estimates for CTDs

For each relative type (full sibling, parents, offspring, all first-degree, and all second-degree), we multiplied the CTD general population prevalence estimate (5) by the reported increased CTD risk to each relative type (18). We also provided the range of risk by multiplying the population prevalence by the 95% CI for CTD increased risk to each relative type. This was done overall as well as separately for each sex.

## Results

Overall, based on general population prevalence and increased risk to relatives, the recurrence risk estimate for CTDs in any first-degree relative is 29.9% (95% CI = 23.2–38.5%) (Table 1). The risk is higher in males, 33.7% (95% CI = 26.2–43.3%), than females, 24.3% (95% CI = 18.9–31.3%). For second-degree relatives, the recurrence risk estimates are considerably lower (7.4%; 95% CI = 5.1–10.4%), also higher in males (8.3%; 95% CI = 5.8–11.7%) than in females (6.0%; 95% CI = 4.2–8.5%).

**Table 1:** Empiric recurrence risk estimate for chronic tic disorders based on general population prevalence and increased risk to relatives.

**Relative type**	**General population prevalence (5)**	**Increased risk to relative OR (95% CI) (18)**	**Recurrence risk % (95% CI)**
**A. OVERALL**			
All 1st degree	1.6%	18.7 (14.5–24.1)	29.9 (23.2–38.5)
Full sibling	1.6%	17.7 (12.9–24.2)	28.3 (20.6–38.7)
Parents	1.6%	21.1 (11.2–39.7)	33.8 (17.9–63.5)
Offspring	1.6%	24.7 (12.4–49.3)	39.5 (19.9–78.9)
All 2nd degree	1.6%	4.6 (3.2–6.5)	7.4 (5.1–10.4)
**B. MALES ONLY**			
All 1st degree	1.8%	18.7 (14.5–24.1)	33.7 (26.2–43.3)
Full sibling	1.8%	17.7 (12.9–24.2)	31.9 (23.2–43.6)
Parents	1.8%	21.1 (11.2–39.7)	38.0 (20.1–71.4)
Offspring	1.8%	24.7 (12.4–49.3)	44.5 (22.4–88.7)
All 2nd degree	1.8%	4.6 (3.2–6.5)	8.3 (5.8–11.7)
**C. FEMALES ONLY**			
All 1st degree	1.3%	18.7 (14.5–24.1)	24.3 (18.9–31.3)
Full sibling	1.3%	17.7 (12.9–24.2)	23.0 (16.8–31.5)
Parents	1.3%	21.1 (11.2–39.7)	27.4 (14.5–51.6)
Offspring	1.3%	24.7 (12.4–49.3)	32.1 (16.1–64.1)
All 2nd degree	1.3%	4.6 (3.2–6.5)	6.0 (4.2–8.5)

## Discussion

For CTDs, the empiric 95% CI recurrence risk estimates for different first-degree relatives (sibling, parents, and offspring) range from 28.3 (sibling) to 39.5% (offspring). The higher population prevalence in males than in females (3–4:1) led to a higher recurrence risk for any first-degree relative in males (33.7% in males vs. 24.3% in females). These recurrence risks estimates are in line with the recurrence risk to offspring of parents with other severe psychiatric diagnoses (schizophrenia, bipolar disorder, major depressive disorder), based on a meta-analysis of high-risk family studies (36). In that study, children of a parent with one of these diagnoses had a 32% chance of having a psychotic or major mood disorder by early adulthood. On the other hand, our CTD empiric recurrence risks are higher than those for schizophrenia and bipolar disorder historically quoted for genetic counselors (37). However, the meta-analysis was published more recently than the genetic counseling quoted risks, and this could explain the difference. In support of the higher risks from the meta-analysis, a recent systematic review of multiple familial high-risk longitudinal studies found that adult offspring of one or more parents with schizophrenia had 15–40% risk of developing a psychotic disorder (38). Thus, the recurrence risks for CTDs are in line with the risks from these other neuropsychiatric disorders.

Our findings should be considered in the context of certain limitations. The prevalence estimate used in this study was from the 2012 meta-analysis by Knight et al. (5). A subsequent meta-analysis, published in 2014, by Scharf et al. (4), included additional studies and had a slightly lower prevalence of TD (Scharf and colleagues' TD prevalence = 0.5%; 95% CI = 0.3–0.9%, compared with Knight and colleagues' TD prevalence 0.8%; 95% CI = 0.4–1.5%). While Scharf and colleagues' article did not report other CTDs, the lower TD prevalence may indicate that the true CTD prevalence and thus the empiric recurrence estimates reported here are slightly lower. On the other hand, a recent meta-analysis of prevalence studies conducted in China, which was not included in either of the prior meta-analyses, found a prevalence of TD plus other CTDs that was similar to the prevalence in the study of Knight et al. (1.5% combined for Chinese study vs. 1.6% in Knight and colleagues' study). Thus, far, there has not been a meta-analysis of family studies. While we used the results from the largest general population family study to date, this is only an estimate of the true underlying increased risk to relatives. Combining multiple family studies would reduce the size of the 95% CI (e.g., offspring 95% CI = 19.9–78.9%). In addition, our reliance on the data from the Swedish national registries to estimate empiric recurrence risk may also be somewhat problematic as the registries overrepresent the more severe or complex cases (18) but may miss less severe cases (that never seek clinical attention) and hence may not generalize to milder forms of the disorder.

In summary, CTDs have a complex heterogeneous etiology that involves hundreds of genes, some with rare high-penetrance mutations and others with common low-penetrance variants that act in an additive fashion. In this article, the empiric recurrence risk estimates range between 30 and 40% for specific first-degree relatives (or all first-degree relatives combined), but the CIs are wide. The recurrence risk estimates for second-degree relatives are considerably lower (7.4%; 95% CI = 5.1–10.4%). The recurrence risks are higher in males than females. These recurrence risks are in line with published risks for other neuropsychiatric disorders, including schizophrenia (36, 38).

### Genetic Counseling Implications

Genetic counseling is a process that helps individuals understand the medical implications, including recurrence risk, for a variety of inherited disorders. The goal of this article was to provide the empiric recurrence risk estimates for CTDs, not the process of how to provide these risk estimates during a genetic counseling session. For an excellent article discussing the process of psychiatric genetic counseling, see Inglis et al. (35).

Previous work has suggested that patients with a family history of psychiatric disorders and other multifactorial disorders are interested in, and benefit from, genetic counseling, despite the challenges and limitations of precise risk assessment (35, 39). It is yet to be explored which families with CTDs might be interested in genetic counseling. However, recent insights into the genetic architecture of CTDs have implications for recurrence risk estimation for families seeking information and thus make genetic counseling for CTDs possible. Presently, genetic counseling for CTDs using the empiric recurrence risks found in this article would follow a comparable model, outlined by Inglis et al. (35), which details an approach for providing psychiatric genetic counseling. Similar to genetic counseling for CTDs, psychiatric genetic counseling often involves providing empiric recurrence risk estimates. That article (35) provides detailed descriptions of (a) exploring personal and family histories, (b) establishing a shared understanding and expectations for counseling, (c) discussing the known and unknown etiology of psychiatric illness, (d) discussing protective factors, (e) communicating risk to patients, and (f) deriving estimates of probabilities for children to develop the illness. Additionally, this article also provides text for clinicians to use for different issues that might arise in a genetic counseling setting [Tables 1–3 Inglis et al. (35)].

Table 2 outlines the key points for couples or patients seeking genetic counseling for CTDs. The key points of a consultation with a genetic counselor include receiving education regarding the hereditary nature of CTDs, understanding its complex and heterogeneous genetic architecture, and comprehending the lack of available genetic testing. Although empiric recurrence risk estimates are available, there is currently no genetic testing that would provide individualized recurrence risk estimates for CTDs or that would rule out any predisposing factor. Genetic counselors are trained to provide a balanced summary of the current understanding of the underlying genetic etiology while highlighting the limitations of such information. Physicians and other medical professionals are encouraged to refer interested parents or patients to genetic counseling. Information surrounding the recurrence risk of CTDs is expected to evolve over time, and genetic counselors are able to provide the most up-to-date information to families at risk.

**Table 2:** Key counseling points for a chronic tic disorder (CTD) genetic counseling appointment.

Prevalence is higher in males than females (3–4:1).
CTDs are highly heritable.
Complex and heterogeneous etiology with hundreds of genes involved, both rare high-penetrance and common low-penetrance variants, and these may interact with environmental factors *in utero*.
Ten percent to 12% of CTDs are due to *de novo* mutations, and these *de novo* mutations in one generation can be passed on to children in the next generation.
Lifetime worst-ever tic severity may be higher when there is at least one other family member is affected, but the number of relatives with tics does not predict severity. Worst-ever tic severity varies widely within family members (i.e., severity does not breed true). Also, there is limited evidence that if both parents are affected, their children will have more severe tics.
At present, genetic testing for Tourette disorder and other CTDs has limited utility.
The empiric recurrence risk estimates for CTD found in this study are an average risk across all family types, including families in which only one individual has a CTD and families in which multiple individuals have had CTD. The 95% confidence intervals show the range of uncertainty around an average value and not meant to imply a range that is clinically applicable to a certain family type. That is, we currently do not have a system to categorize families with higher or lower risk within these intervals.

One aspect that we did not address is the risk to relatives for the associated disorders that are known to be often clinically comorbid with CTDs, such as ADHD or OCD, but that may be at increased risk even in offspring without tics (10, 25). Furthermore, because genetic counseling is often sought in the context of couples contemplating conceiving in the context of a family history of CTD, preconceived notions about the quality of life with CTD may affect such decisions and be heavily colored by the specific experiences of affected family members. The quality of life over the life span of individuals with CTDs varies greatly with their clinical specifics, especially the presence or absence of comorbidities such as ADHD or OCD, age, family support, and treatment (40, 41). Although these are not the purview of the genetic counselor *per se*, it is important that individuals and families with CTD be connected with clinicians with expertise in these disorders who can help to mitigate their potentially deleterious impact.

## Data Availability Statement

All datasets generated for this study are included in the article.

## Ethics Statement

Ethical approval and written informed consent were not required as per local legislation and national guidelines.

## Author Contributions

GH, JR, CS, JL, RK, and TF contributed to the design, organization, and conception of the work. GH and TF contributed to the statistical analysis. GH, JR, and TF contributed to the interpretation of the data. GH, JR, and RK contributed to drafting the manuscript. All authors reviewed, critiqued, and gave final approval for the version to be published and agreed to be accountable for all aspects of the work.

## Conflict of Interest

The authors declare that the research was conducted in the absence of any commercial or financial relationships that could be construed as a potential conflict of interest.
